# Vibration or Stretch? Distinct Mechanoelectrical Signatures Govern Osteogenic Programming in PVDF

**DOI:** 10.1021/acsami.5c23327

**Published:** 2026-02-09

**Authors:** Sylvie Ribeiro, Clarisse Ribeiro, Nélson Castro, Vitor Correia, Igor Irastorza, Unai Silván, Senentxu Lanceros-Mendez

**Affiliations:** † CF-UM-UP − Physics Centre of Minho and Porto Universities and LaPMET − Laboratory of Physics for Materials and Emergent Technologies, 56059 University of Minho, Braga 4710-057, Portugal; ‡ Algoritmi Research Centre, University of Minho, Campus de Azurém, Guimarães 4800-058, Portugal; § SYSTEC− Research Center for Systems and Technologies- University of Porto (FEUP), Porto 4200-465, Portugal; ∥ Faculty of Engineering, University of Porto, FEUP, Porto 4200-465, Portugal; ⊥ Cell Biology and Histology Department, University of the Basque Country (UPV/EHU), 48940 Leioa, Spain; # BCMaterials, Basque Centre for Materials, 518636 Applications and Nanostructures, UPV/EHU Science Park, Leioa 48940, Spain; & Basque Foundation for Science, 197447 Ikerbasque, Bilbao 48009, Spain

**Keywords:** bone tissue engineering, piezoelectric scaffolds, mechanoelectrical stimulation, cyclic stretching, vibration bioreactor, osteogenic differentiation, calcium signaling

## Abstract

A promising method for directing cell behavior and tissue regeneration is the use of smart materials that can transform physical inputs into bioelectrical signals. In this study, the mechanoelectrical control of preosteoblast activity was investigated using a piezoelectric smart biointerface based on positively poled poly­(vinylidene fluoride) (PVDF). Distinct mechanical regimes, including vibrational and cyclic stretching, were applied through customized bioreactors, enabling controlled mechanoelectrical inputs ranging from 63 to 227 μVpp mm^–2^. The biological response of MC3T3-E1 cells was evaluated in terms of metabolic activity, intracellular calcium signaling, alkaline phosphatase (ALP) activity, matrix mineralization, and gene expression (RUNX2, ALP, OPN, and OCN). The results demonstrated that stretching stimulation combined with higher mechano electric inputs (113–227 μVpp mm^–2^) enhanced calcium influx and enhanced osteogenic differentiation, while lower impulses (∼63 μVpp mm^–2^) under vibrational circumstances increased cell proliferation. These findings highlight the intensity- and mode-dependent nature of mechanoelectrical signaling in regulating osteogenic commitment. All things considered, this study shows how piezoelectric smart materials can be used as bioresponsive platforms to precisely control cell proliferation and differentiation, creating avenues for bone tissue engineering’s next-generation regenerative techniques.

## Graphical abstract

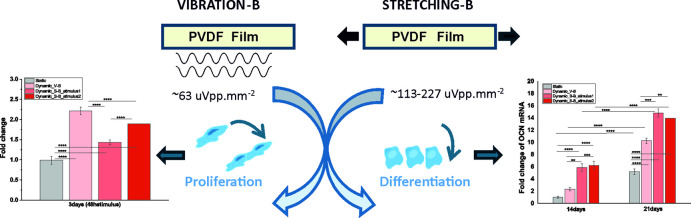

## 1 Introduction

Bone fractures are partial or total breaks in the bone that can occur spontaneously (due to diseases like osteoporosis and related chronic conditions) or as a result of trauma or falls (e.g., sports injuries or car accidents).^1^ Fractures are linked to considerable morbidity, mortality, and healthcare costs, making them a global public health concern.^2^ Fractures can lead to work absence, reduced productivity, disability, impaired quality of life, health loss, and high health-care costs, thus placing a major burden on individuals, families, societies, and health-care systems.^3^

A growing number of patients are experiencing disease-related or trauma-related bone tumor resections, fracture defects, or persistent infections, and over 1.5 million bone transplant procedures are carried out annually in the United States, making it, after blood, the second most often transplanted tissue.^4^ Autograft is the gold standard for bone healing when treating nonunion and bone abnormalities. Autografts do have several drawbacks, though, including a restricted supply, fresh nerve injury, lingering discomfort, and new fractures. Since the allograft has a plentiful supply and excellent osteoconductivity, it has been employed successfully in orthopedic procedures. Allografts do, however, carry a risk of infection, disease transmission, and an immunological reaction. However, because allografts need to be processed, sterilized, and preserved before being utilized, they are less effective at encouraging bone regeneration than autografts.^5^

In this context, tissue engineering (TE) offers a potential method for bone repair.^6,7^ In addition to offering mechanical strength, a place for vascularization and tissue infiltration, and the ability to be both osteoinductive and osteoconductive, the perfect bone grafting material should also operate as a carrier for pertinent therapeutic elements.^8,9^

Advancements in bone tissue engineering have led to the development of biomaterials, bioactive molecules, and biophysical stimulation strategies to enhance bone regeneration,^10,11^ with recent approaches increasingly integrating piezoelectric signals to actively regulate osteogenesis and functional tissue regeneration.^12^ A key focus has been the use of scaffolds mimicking the natural extracellular matrix (ECM), thereby supporting cell adhesion, proliferation, and differentiation.^13–15^ Various biomaterials have been explored, including natural polymers (e.g., collagen, chitosan), synthetic polymers (e.g., polycaprolactone, polylactic acid), and bioceramics (e.g., hydroxyapatite, tricalcium phosphate).^16–19^

In addition to biochemical cues, biophysical stimulation, including mechanical loading and electrical signals, play a crucial role in modulating osteogenic differentiation,^20,21^ within a mechanobiological framework that integrates physical forces and cellular responses.^22^ Bone is a piezoelectric tissue, meaning it generates electrical potentials in response to mechanical deformation, which, in turn, influence cell behavior.^23,24^ Therefore, piezoelectric biomaterials, particularly poly­(vinylidene fluoride) (PVDF), have emerged as promising candidates for bone tissue engineering, due to their ability to provide mechanoelectrical stimuli that mimic the natural bone biophysical active microenvironment.^25–27^

Recent studies have demonstrated that mechanical loading enhances osteoblast activity and mineralization by promoting intracellular calcium signaling, upregulating osteogenic markers (RUNX2, ALP, OPN, and OCN), and increasing matrix deposition.^28–30^ Different types of bioreactors, including compression, tensile strain (stretching), vibration, and perfusion systems, have been developed to replicate mechanical stimuli under controlled conditions.^31–33^ In particular, studies have shown that low-intensity vibrational stimuli favor cell proliferation,^34^ whereas tensile strain accelerates osteogenic differentiation.^35^ Furthermore, mechanoelectrical cues, in combination with biochemical factors such as growth factors (e.g., BMP-2, VEGF, FGF) or bioactive ions (e.g., Ca^2+^, Mg^2+^, Sr^2+^), can enhance bone regeneration outcomes.^36,37^ Despite this progress, the specific impact of different mechanical and mechanoelectrical stimulation intensities on osteogenic differentiation remains insufficiently explored. Additionally, the combined impact of mechanical stimulation modality (vibration vs stretching) and electrical input applied through piezoelectric scaffolds merits further investigation.

Considering the importance of understanding the combined impact of intensity and type of mechanical and electrical stimulation, this study aims to specifically evaluate the effect of different mechanoelectrical stimulus intensities applied through vibrational and stretching bioreactors using piezoelectric PVDF substrates. This will help to identify and understand optimal conditions favoring cell proliferation and osteogenic differentiation, thereby contributing to the design of more effective bone tissue engineering strategies.

## 2 Experimental Section

### 2.1 Substrate Preparation

β-Phase PVDF films (PVDF-P0100, PolyK) with different surface charges (average none “PVDF NP”, positive “PVDF P+”, and negative “PVDF P–”), with a thickness of 110 μm, were used. The materials used present a |d_33_| and |d_31_| response of 25 and 30 pC N^–1^, respectively, and a dielectric constant of ∼12.5. Both materials present a similar degree of crystallinity (46%),^38^ surface roughness (42 nm)^39^ and a value of contact angle of 83.1° ± 2.2° and 51.3° ± 3.1° for PVDF NP and PVDF P+, respectively.^40^

Three replicates of the different PVDF films were cut and sterilized under ultraviolet (UV) light for 30 min on each side before being washed twice with phosphate-buffered saline (PBS). Disks 13 mm in diameter were cut and placed on 24-well culture plates for static condition and dynamic condition with a vibration bioreactor (V-B), and rectangles with 1 cm × 3 cm diameter were cut for the stretching bioreactor (S-B). All the results were normalized per cm^2^.

### 2.2 Cell Culture Assays

#### 2.2.1 Preosteoblast cell culture

MC3T3-E1 cells (Mouse-Riken, passage number: 25–30) were grown in 75 cm^2^ cell-culture flask and cultured in basal medium (BM), consisting of Dulbecco’s Modified Eagle’s Medium (DMEM, PAN-Biotech) containing 1 g L^–1^ supplemented with 10% Fetal Bovine Serum (FBS, PAN-Biotech) and 1% Penicillin-Streptomycin (P/S, Biochrom). The flask was incubated at 37 °C in a humidified incubator containing a 5% CO_2_ atmosphere. The culture medium was exchanged every 2 days and when the cells reached 60%–70% confluence, they were trypsinized with 0.05% trypsin-EDTA and subcultured.

For the experiments, MC3T3-E1 cells were seeded on control (polystyrene) and the poly­(vinylidene fluoride) poled positively (PVDF P+) films at a density of 15.000 and 30.000 cells/cm^2^ for proliferation and differentiation tests, respectively. The drop method was used for the proliferation and differentiation assays with the S-B.

For cell proliferation studies, three plates under these conditions were incubated with BM at 37 °C in a saturated humidity atmosphere containing 95% air and 5% CO_2_ for 24 h to allow cell adhesion. After this time, one plate was maintained at the same conditions (static culture, meaning the cell culture without any applied stimuli) and the others were transferred onto the V-B^41^ and the S-B systems for dynamic culture - meaning the cell culture under mechanical stimulation for mechanoelectric response - up to 3 days.

For the differentiation experiments, the samples were placed in 24-well plates (two plates) and one plate with 4 wells, and incubated in BM, as previously described, until reaching 90% confluence. Thereafter, to induce osteogenic differentiation, the BM was replaced with the differentiation medium (DM). The DM was composed of DMEM supplemented with 10% FBS and 1% P/S supplemented with 50 μg mL^–1^ ascorbic acid, 10 mM β-glycerophosphate and 0.1 μM dexamethasone. As for the proliferation assay, one plate was maintained under the same conditions (static culture), and the other ones were transferred onto the V-B and the S-B for dynamic culture up to 21 days. In all conditions, the medium was renovated every 2 days.

For dynamic culture, two different bioreactor systems were used: a vibrational bioreactor (V-B) and a stretching bioreactor (S-B). In both setups, samples were subjected to the same mechanical stimulation protocol, consisting of two 1 h sessions per day, separated by a 5-h interval. In the stretching bioreactor, mechanical strain was applied through the controlled displacement of the clamping system. In the vibrational bioreactor, the culture plate was placed on a vibrating module operating at a frequency of 1 Hz and an amplitude of approximately 1 mm.^42^

Four replicates were used for each condition. Figure 1 presents the experimental design of the study.

**Figure 1:**
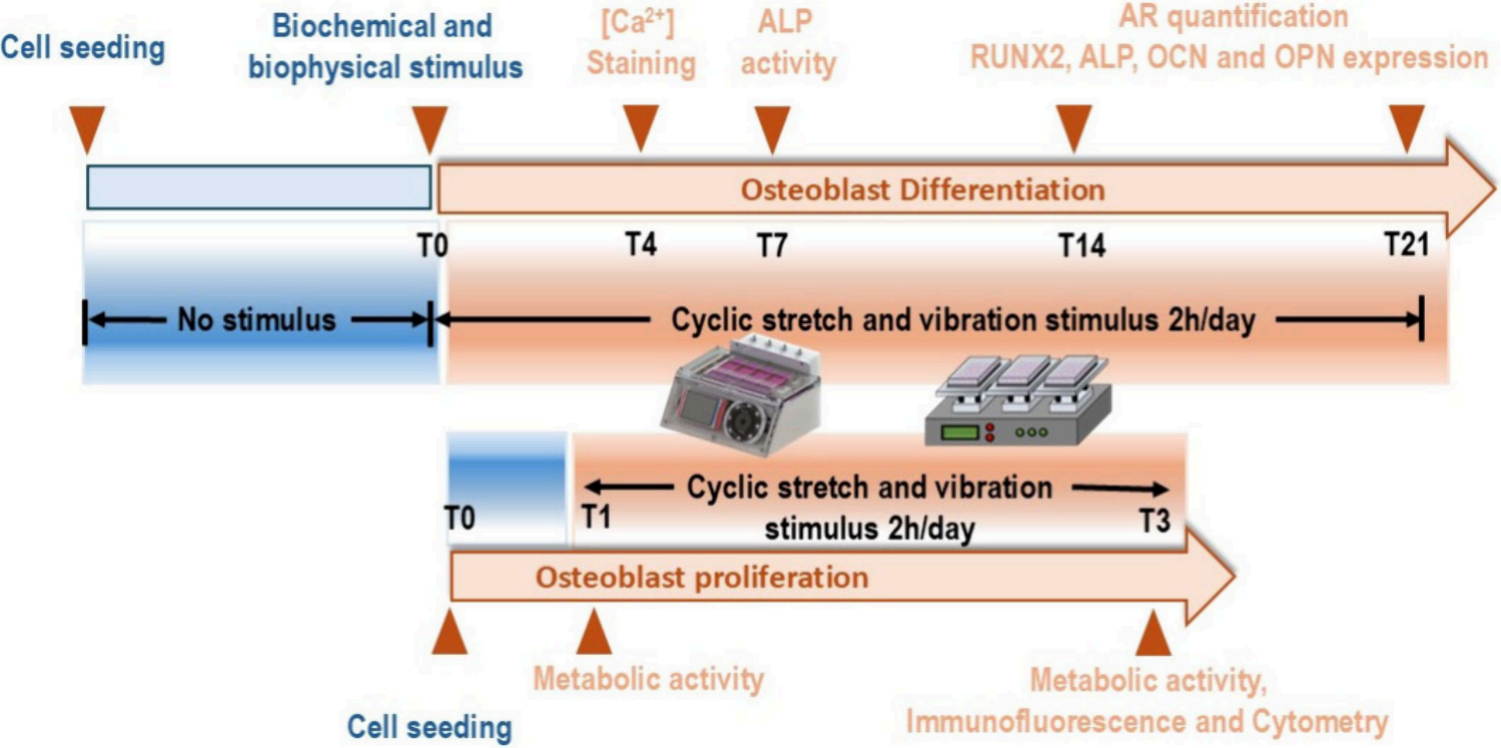
Schematic timeline of the experimental design used in this study. The upper panel represents the osteogenic differentiation protocol, where MC3T3-E1 cells were exposed to cyclic mechanical stimuli (stretching or vibration) for 2 h per day, starting at T0 (after reaching 90% confluence) and continuing until day 21. Key assessment time points included intracellular calcium staining (T4), ALP activity (T7), and mineralization/osteogenic marker expression (T14–T21). The lower panel represents the proliferation assay timeline, with mechanical stimulation applied from T1 to T3, and analysis performed at T1 and T3 for metabolic activity, immunofluorescence, and cytometry. Both static (no stimulus) and dynamic conditions were evaluated.

#### 2.2.2 Cell Culture Characterization

##### 2.2.2.1 Cell Viability Assay

After 3 days of culture, viable cells were measured using a CellTiter 96 Aqueous One solution Cell proliferation assay ((3-(4,5-dimethylthiazol-2-yl)-5-(3-carboxymethoxyphenyl)-2-(4-sulfophenyl)-2H-tetrazolium), MTS, Promega). For that, cells were incubated in an MTS-DMEM low-glucose medium (1:5, respectively) solution for 3 h at 37 °C, 5% CO_2_ and 95% humidified air atmosphere. The absorbance was measured at 490 nm with a microplate reader (Biotech Synergy HT, Winooski, VT, USA), and cell proliferation was determined regarding the cells adhered on the material after 24 h of adhesion, just before placing them in contact with the bioreactor (eq 1). The obtained results are shown as the mean ± standard error of the mean (SEM) of triplicate samples.

1 $$\mathrm{cell proliferation (\%)}=\frac{{\mathrm{Abs}}_{\mathrm{490 nm}}\;\mathrm{sample after 72 h cell culture}}{{\mathrm{Abs}}_{\mathrm{490 nm}}\;\mathrm{sample after 24 h cell culture}}\times 100$$

##### 2.2.2.2 Immunofluorescent Staining

After removal of the medium from each well, the cells were fixed with 4% formaldehyde for 10 min at 37 °C and rinsed with PBS 1× to prepare for immunofluorescence imaging. The samples were then treated with 0.1 μg mL^–1^ FITC phalloidin (Sigma–Aldrich) for 45 min at room temperature to stain the actin cytoskeleton. After three washes with PBS once, the samples were incubated for an additional 5 min with 1 μg mL^–1^ DAPI to stain the nuclei. Following three more PBS washes, the samples were carefully taken out of the wells, mounted on slides, and visualized using a fluorescence microscope (Olympus, Model BX51) equipped with the corresponding filter sets for the used fluorophores.

##### 2.2.2.3 Cell Cycle Analysis

Flow cytometry was used to assess propidium iodide (PI) staining using a cell cycle analysis kit (MAK344, Sigma–Aldrich). Adherent cells were collected, pelleted, washed, and then fixed in 70% ethanol for 30 min at 4 °C. After centrifugation, the cells were stained with PI solution according to the manufacturer’s instructions and incubated for 30 min at room temperature in darkness. PI-stained cells were analyzed using a flow cytometer (Cytoflex), acquiring at least 10 000 events per sample. Cell cycle distribution was determined using FlowJo software (version 10, Tree Star, Inc., San Francisco, CA, USA), based on at least four independent experiments.

##### 2.2.2.4 Fluo-4 AM Intracellular Calcium Staining

Fluo-4 is the most popular green fluorescent intracellular calcium (Ca^2+^) indicator. Initially, the solution was prepared by adding 10 mL of assay buffer (HEPES-buffered Hank’s Balanced salt solution, pH 7.2–7.4) to a conical tube, 100 μL of 100X Pluronic F-127 solution ensuring equitable dye distribution and cellular loading, and vortexing briefly the tube to mix the components. After that, the Fluo-4 AM (50 μg) was dissolved in 25 μL of DMSO, vortexed to dissolve the indicator dye and put this content to assay buffer solution (referred above) to prepare the working solution. Then at the specified time point, the cell culture medium was removed, and the dye loading solution was added to the wells (500 μL per well in a 24-well plate) and incubated for 60 min at 37 °C. Fluorescence is measured (Ex: 480 nm/Em: 515 nm) with a microplate reader (Biotech Synergy HT, Winooski, VT, USA) and parallelly visualized using a fluorescence microscope (Olympus, Model BX51) equipped with the corresponding filter sets for the label used.

##### 2.2.2.5 Alkaline Phosphatase Assay

According to the procedure described in ref 43, alkaline phosphatase (ALP) activity was quantified after 7 days of MC3T3-E1 osteogenic differentiation under static and dynamic conditions. Initially, the cells were lysed with 0.1% of Triton buffer solution (Sigma–Aldrich), collected and frozen at −80 °C. After freezing, *p*-nitrophenyl phosphate and 2-amino-2–2-methyl-1-propanol were added in a 1:1 ratio, following the manufacturer’s instructions (ALP, Sigma–Aldrich). The amount of produced *p*-NP (*p*-nitrophenol) was quantified by using a microplate reader (BioRad Lab) at 405 nm. To normalize the ALP activity results, the total was quantified from the cell lysate using a CyQUANT Cell Proliferation Assay Kit (Life Technologies), in accordance with the manufacturer’s protocol. Then, the fluorescence of each sample was measured by exciting the sample at 480 nm and measuring the emission at 520 nm using a Biorad Lab reader.

Normalized ALP activity values were expressed relative to those of untreated control cells cultured on PVDF P+ under static conditions.

##### 2.2.2.6 Alizarin Red Staining and Quantification

The gold standard for measuring osteoblast mineralization is the alizarin red staining (ARS, Catalog No. A5533, Sigma–Aldrich) assay. ARS analysis was performed at 14 and 21 days of osteogenic differentiation, as calcium mineral deposition in MC3T3-E1 cells is not expected at earlier time points, such as 7 days, which are primarily associated with premineralization events. Briefly, after 14 and 21 days of osteogenic differentiation under static and dynamic conditions, cells were fixed with 4% formaldehyde in PBS for 10 min at 37 °C. After that, they were washed three times with distilled water and incubated with ARS solution (40 mM, pH 4.2) for 30 min at room temperature under gentle agitation. After staining, samples were rinsed 3 times in distilled water and examined using an Olympus BX51 fluorescence microscope. For quantification, the stained samples were incubated with a 10% (v/v) acetic acid solution for 30 min at room temperature under agitation. Cells were then scraped, vortexed for 30 s, and incubated at 85 °C for 10 min. Next, samples were placed on ice for 5 min and centrifuged for 15 min at 20.000 g. Then, the supernatant was neutralized with 10% (v/v) ammonium hydroxide. Finally, 50 μL of each sample/standard was transferred to an opaque-walled, clear-bottom 96-well plate, and the absorbance was measured spectrophotometrically at 405 nm. Quantitative analysis was then carried out using the standard curve.

All quantitative results were expressed relative to untreated control cells cultured on PVDF P+ under static conditions.

##### 2.2.2.7 Quantitastive Polymerase Chain Reaction

RNA was extracted from cells cultured on the control and piezoelectric materials under the experimental conditions described in Section 2.2.1 (differentiation culture at 14 and 21 days as time points) by adding a solution of TRIzol (Catalog No. 15596026, Invitrogen) and chloroform (Catalog No. C0549–1PT, Sigma–Aldrich) at a 5:1 ratio. The samples were readily dissolved in this solution by gentle pipetting for 2–3 min at room temperature. Then, they were centrifuged for 15 min at 12 000*g* and 4 °C to obtain organic and aqueous phases. The upper aqueous phases were collected, and RNA was precipitated by adding an equal volume of isopropanol. The solution was incubated for 10 min at 4 °C. Thereafter, samples were centrifuged again for 10 min at 12 000*g* and 4 °C, when precipitated RNA formed a pellet that was washed with a 75% ethanol solution and centrifuged again for 5 min at 7500 *g* at 4 °C. After that, resuspended 20 μL of RNase free water, treated with DNase and put in a heat-block at 55–60 °C between 10 and 15 min. RNA purity and concentration were calculated using an Infinite M Nano+ plate reader (Tecan) at 260/280 nm. Reverse transcription reaction was performed using iScript cDNA Synthesis Kit (Biorad). RT-qPCR was performed using a QuantStudio 1 thermocycler (from Applied Biosystems) and the SYBR Green method. The primers for amplification are given in Table 1. The amplification protocol used consisted of an initial step at 95 °C for 3 min, followed by 42 cycles at 95 °C for 15 s and at 60 °C for 30 s.

**Table 1:** Forward and Reverse Primers for the Amplification of the Analyzed Genes

gene	differentiation stage	primer sequence (5́-3́)		Tm	length	amplicon size (bp)
Rn18S	Housekeeping	Forward	GGCCGTTCTTAGTTGGTGGA	59.96	20	148
		Reverse	CTCAATCTCGGGTGGCTGAA	59.75	20	
						
RUNX2	Early	Forward	TCAGTGAGTGCTCTAACC	59.75	18	155
		Reverse	TGCCTGGAGTACATAGAC	59.94	18	
						
ALP	Early/Intermediate	Forward	GACACAGACTGCACAGAT	59.89	18	102
		Reverse	GGAGAGAAGGTCAGATCT	59.82	18	
						
OPN	Intermediate/Late	Forward	AGTCCCTCGATGTCATCCCT	59.74	20	160
		Reverse	GACTGATCGGCACTCTCCTG	59.90	20	
						
OCN	Late	Forward	GCTTAACCCTGCTTGTGACG	67.0	20	146
		Reverse	GATCAAGTCCCGGAGAGCAG	64.4	20	

Gene expression quantification was performed using comparative 2^–ΔCT^, with Rn18s as a housekeeping gene. The results are expressed as mean ± standard error of the mean (SEM).

Relative gene expression was calculated by using the untreated static PVDF P+ condition as the biological control.

### 2.3 Statistical analysis

Statistical analysis for all tests was performed using the GraphPad Prism program (v9.2.0, GraphPad, La Jolla, CA, USA) with one-way ANOVA and two-way ANOVA followed by tukey’s multiple comparisons test. Differences were considered statistically significant when the *p*-value is <0.05.

## 3 Results and Discussion

### 3.1 Characterization of the Mechanoelectrical Response of the Bioreactors

#### 3.1.1 Characterization of the Stretching Bioreactor (S-B) and Mechanoelectrical Stimulation Measurement

A custom-made bioreactor capable of applying controlled deformations was used (Figure 2a). This equipment is characterized by allowing the use of commercial culture plates with 4 wells (Figure 2b), which simplifies the operation and sterilization process of the surfaces that are in contact with the cells. The application of the mechanical stretching force on the piezoelectric polymer substrates is obtained through a magnetic link with the substrate grippers. This solution presents numerous advantages in terms of simplifying the entire operation process and sterilizing the surfaces; however, it presents a maximum force limited by the magnetic attraction between the magnet of the moving body within the bioreactor and the magnet of the moving claw stretching the samples. The applied force was measured by connecting a 10 N load cell to the moving claw and applying the displacement to the novel body of the bioreactor in both directions. The maximum applied force in the experiments was around 1 N, allowing polymer deformations up to 25 μm, as shown in Figure 2d.

**Figure 2:**
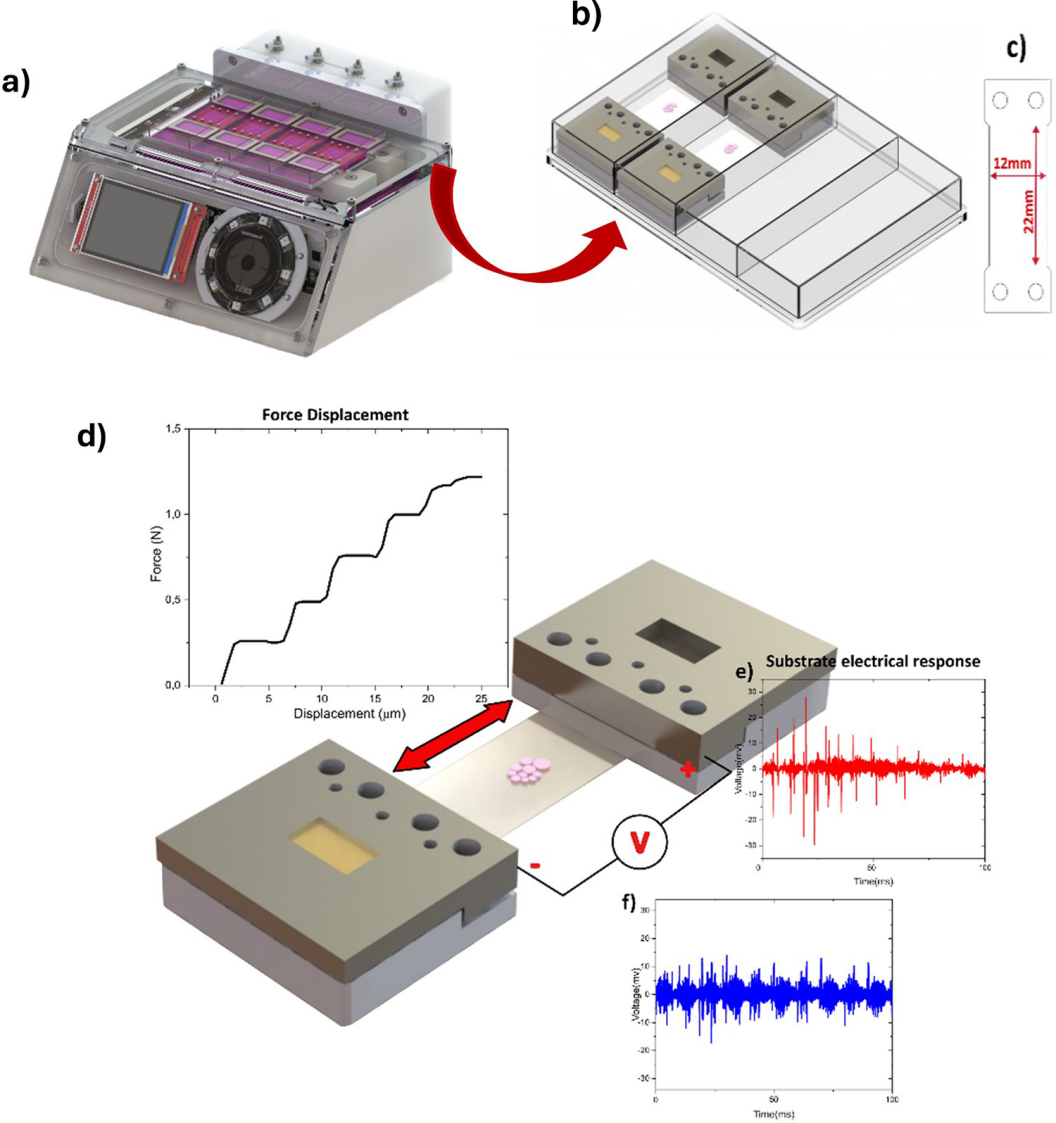
(a) Stretching bioreactor used in the tests performed in this study; (b) claw system with magnetic links; (c) dimensions of the flexible substrate used in the culture process suitable for fitting in the claws; (d) single curve of force vs stretching displacement of the substrate material, and (e, f) respective electrical response between claws in response to a deformation pulse ((e) 0.025 mm (red) and (f) 0.015 mm (blue)).

For the tests, the 4 culture wells were used simultaneously to obtain replicates of the cell culture. Piezoelectric poly­(vinylidene fluoride) was used as the substrate, with the specific shape and dimensions (Figure 2c) compatible with the shape of the magnetic claws and the corresponding fixing holes, as shown in Figure 2b.

To close the clamp, the upper magnetic clamp is placed, which prevents the substrate from being loose during the stimulation process.

The study of mechanical deformation of the samples within a culture system (see Figure S1 in the Supporting Information) was performed through stress–strain curves of the cell culture tests. For the tests, the universal testing machine (Shimadzu-AG-IS_500 N load cell) was used, and the deformation behavior of a material under uniaxial tensile load was recorded. Analyzing this response, it is possible to conclude that the material used presents an elastic region that goes far beyond the deformations produced by the bioreactor. In this case, the zone up to the maximum force applied to the sample was analyzed. Thus, it was verified that the system will apply an approximate deformation of 10 μm.

Considering the piezoelectric characteristics of the materials used as culture substrates, their electrical response to two different mechanical deformation amplitudes was analyzed, showing a proportional reduction in the electrical response with decreasing mechanical amplitude, corresponding to the direct piezoelectric response of the material. A 16-bit oscilloscope (PicoScope 5000) was used, connected via USB and operated with the PicoScope 7 software to record the electrical response. A test lead with a 1 MΩ resistance was placed between the two clamps, securing the cultivation substrate, enabling the measurement of the potential difference generated between them. The test was conducted under the same conditions as the cellular experiments, with a frequency of 0.3 Hz and deformations of 0.025 and 0.015 mm.

When exposed to mechanical deformation due to its piezoelectric characteristics, the material used generates a potential difference corresponding to an electrical stimulus that can affect the cells. Given the pulsed profile of the applied deformation, the potential difference also corresponds to a pulsed response. Analyzing the electrical response of the material to mechanical deformation, this reflects the typical response of a piezoelectric material when subjected to force variations. In the operating mode of the system, which is based on displacement steps, the electrical response occurring just during moments of force or deformation change, as shown in Figure 2e and 2f. A peak-to-peak variation of more than 30 mV for a deformation of 0.015 mm and 60 mV for 0.025 mm was obtained between the clamps, which translates, according to the area of the substrate used, into an electrical response per unit area of 113 μVpp mm^–2^ (stimulus 1) and 227 μVpp mm^–2^ (stimulus 2), respectively. This response is consistently repeated every 100 ms, corresponding to the interval at which the bioreactor applies the displacement to the substrate.

#### 3.1.2 Characterization of Electrical Response of Vibration Bioreactor (V-B)

The electrical response of the vibrational bioreactor (Figure 3a)^42^ was characterized by a piezoelectric sample with conductive electrodes on both faces of the piezoelectric polymer, allowing for the collection of the electrical signal.

**Figure 3:**
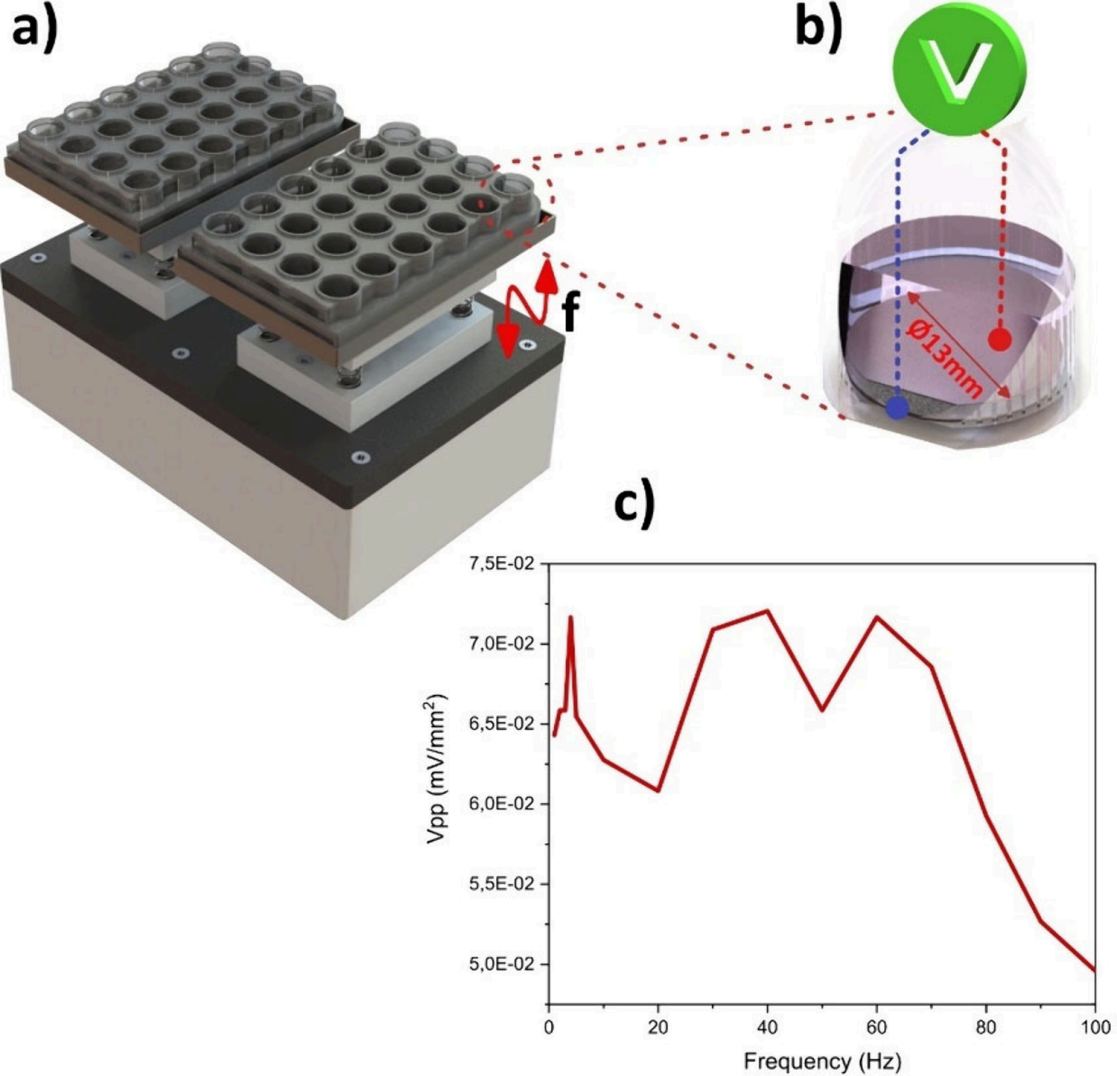
(a) Representative image of the vibration bioreactor (V-B) used in this study; (b) schematic illustration of the method used to measure the electrical response of the scaffolds under mechanical stimuli; and (c) mechanoelectrical response of the sample as a function of applied vibration frequency.

To measure the piezoelectric response of the sample under mechanical stimulation, we applied a frequency spectrum provided by the bioreactor was applied. The sample was connected to a precision and low-noise amplifier circuit with a gain of 1000. The circuit output was then connected to a high-resolution 16 bit oscilloscope (PicoScope 5242D) to record the electrical response in the time domain.

The electrical response of the standard sample was recorded for mechanical excitation frequencies of 1 to 100 Hz.

The electrical response at the sample terminals (Figure 3b and 3c) reveals the periodicity of the mechanical stimulus (1 Hz). A nonlinearity in the electrical response was observed, possibly due to the resonance variation of the mechanical architecture of the bioreactor.

An average electrical response of 67 μVpp mm^–2^ was recorded for frequencies up to 70 Hz, with a decay above this frequency, attributed to the vibration constraints of the bioreactor at higher frequencies. Considering the electrical response of ∼63 μVpp mm^–2^ for the frequency used in cell cultures (1 Hz) (Figure 3c) and the scaffold diameter of 13 mm, a total electrical response of 8.53 mVpp was obtained.

### 3.2 Cell Response to Piezoelectric Poly­(Vinylidene Fluoride) with 2 Different Types of Stimuli

Surface topography and chemical composition modulate protein adhesion and consequently affect cellular behavior.^44^ Thus, controlling the morphology of scaffolds is important to assess the contribution of surface charge to the cell behavior. In fact, it was already proven that poly­(vinylidene fluoride) promotes preosteoblast growth, namely with a slight increase when in contact to the positive surface charge.^45^ Given that bone is a piezoelectric tissue, it exhibits electrically responsive properties that regulate various cellular functions, including morphology, proliferation, and osteogenic differentiation, by electrical signals. Thus, reproducing the piezoelectric microenvironment of bone by applying mechanical cues representative of daily physical activity is crucial. In this study, MC3T3-E1 preosteoblasts were cultured on PVDF substrates and subjected to two distinct types of mechanical stimulation: vibration (used as reference) and stretching (a novel approach), both applied using custom-made bioreactors. Bioreactors allow one to mimic physiological mechanical inputs while enabling a comparative evaluation of how different mechanoelectrical stimuli affect cellular proliferation and osteogenic differentiation.

#### 3.2.1 MC3T3-E1 Response to the Stimulation Protocols

Previous studies have reported that mechanical deformation induces electric polarization, which has been shown to affect the proliferation of MC3T3-E1 cells.^46^ To determine the ability of neat PVDF films with different surface charges and high d_31_ and d_33_ coefficients to support bone cell adhesion and proliferation, preosteoblast cells were cultured on all film types for 3 days under both static and dynamic condition (vibrational bioreactor) (Figure 4).

**Figure 4:**
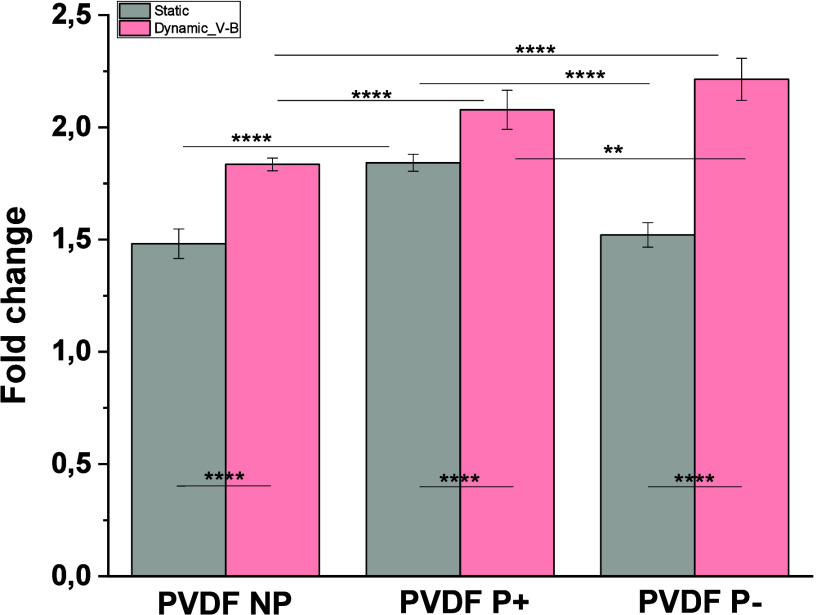
Metabolic activity of MC3T3-E1 cells cultured in neat PVDF with different surface charge at different time points under static and dynamic conditions. The stimulus from vibrational bioreactor at 2 h/day with 1 Hz during 48 h. Graph bars are represented as mean ± SEM. Significance values: (**) *p* < 0.0021 and (****) *p* < 0.0001.

Regardless of the surface charge, the vibration-based stimulation protocols promoted cellular metabolic activity, as assessed by fluorescence-based assays. However, a more pronounced effect was observed in the charged films, with significant differences between both polarizations, particularly under static conditions, where PVDF P+ showed the most favorable results. Based on these results and supported by previous studies demonstrating its superior performance with MC3T3-E1 cells,^47,48^ the positively poled PVDF (PVDF P+) was selected for all subsequent experiments. The mechanical component of the vibration-based protocols may have played a relevant role in the observed enhanced cellular activity observed. Vibrational stimulation provides high-frequency, low-amplitude mechanical cues that have been shown to activate mechanosensitive pathways while minimizing large-scale deformation of the cell membrane. This type of mechanical input has been associated with the promotion of cell proliferation and metabolic activity in osteoblast-lineage cells, as reported in previous studies using related vibration systems.^46^

Given the piezoelectric nature of bone and disorders relating to it, piezoelectric scaffolds that are electromechanically stimulated allow the necessary mechanoelectrical dynamic microenvironment for the efficient growth and differentiation of bone cells. Figure 5 presents the cell viability comparing the static culture conditions and the 2 types of dynamic mechanoelectrical stimuli (vibration and stretching). Furthermore, for the stretching bioreactor (S-B), two different cell culture conditions were applied. A qualitative analysis of the cells stained with FITC and DAPI is also shown to evaluate the cell morphology in all studied cases.

**Figure 5:**
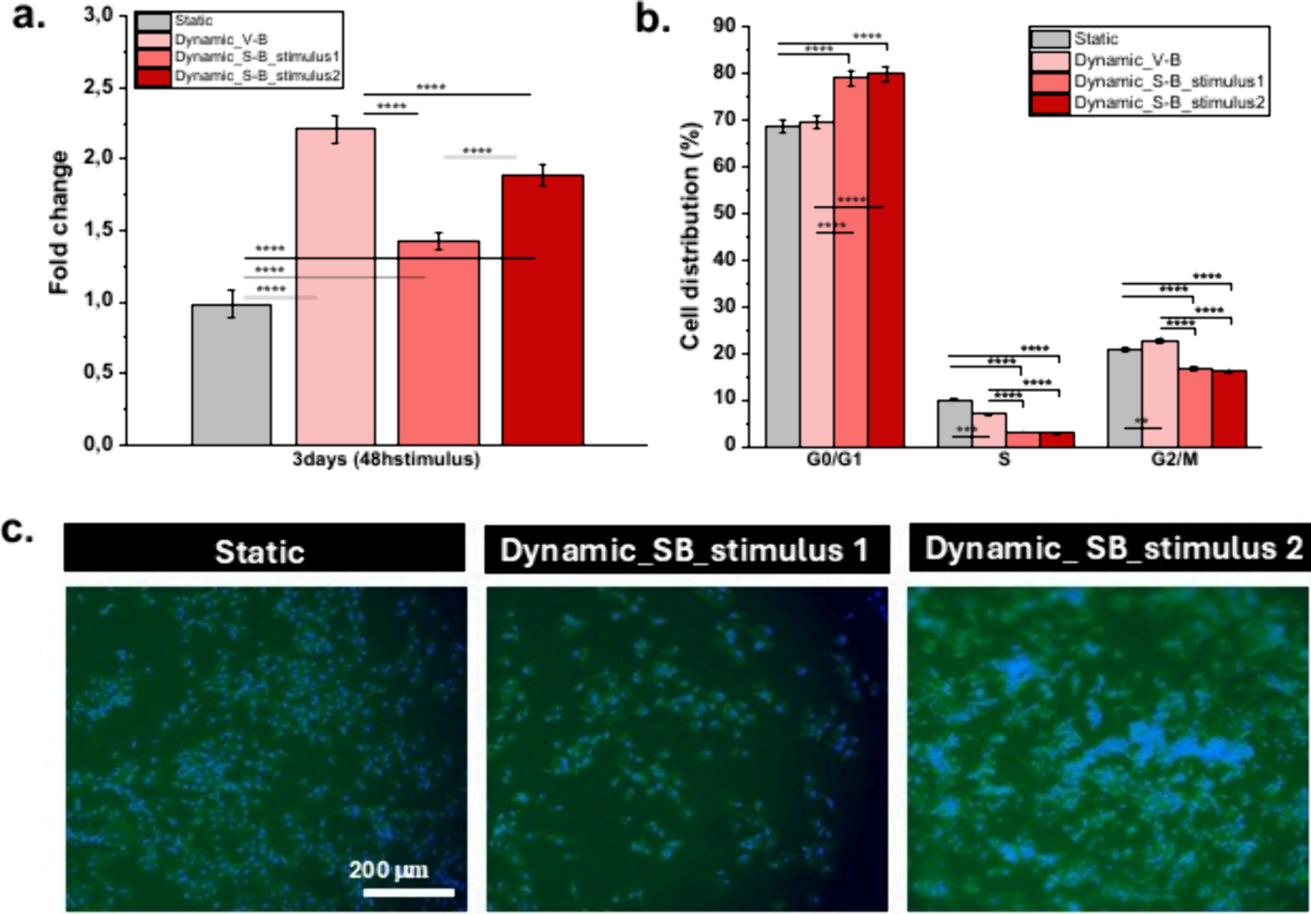
(a) Metabolic activity of MC3T3-E1 cells cultured in PVDF P+ films after 72 h with 48 h of stimulus. All data are normalized to untreated cells cultured on PVDF P+ under static conditions. (b) The cell cycle changes for MC3T3-E1 preosteoblast under static and dynamic conditions for 72 h using flow cytometry. G0/G1 phase: cell growth and preparation for DNA synthesis; S phase: DNA synthesis; G2/M phase: preparation for mitosis and cell division. (c) Immunofluorescence images. Scale bar = 200 μm and is valid for all the images. Graph bars represent mean ± SEM. Significance values: (**) *p* < 0.0021; (***) *p* < 0.0002 and (****) *p* < 0.0001 (two-way ANOVA with a Tukey’s multiple comparisons test, *n* = 4).

Dynamic conditions promote the metabolic activity of the cells, demonstrating differences between all the samples submitted with the different mechanoelectric stimulus (Figure 5a). Under static conditions, PVDF P+ showed a 1-fold increase in cell activity compared to PVDF P+ at 24 h, corresponding to 2.2-, 1.4- and 1.9-fold increases for mechanoelectric stimuli of 63 (dynamic V-B), 113 (dynamic S-B, stimulus 1), and 227 (dynamic S-B, stimulus 2) μVpp mm^–2^, respectively. Interestingly, the best osteogenic behavior was obtained with the lower stimulus, confirming results previously reported in the literature for human adipose stem cells using a vertical vibration module.^47^ When a stretching bioreactor was used, the metabolic activity associated with cell proliferation was significantly lower, being higher for the 227 μVpp mm^–2^ stimulus, compared to the 113 μVpp mm^–2^ one. These results are consistent with previous findings by Zhang et al., who reported that a surface potential of approximately −53 mV enhanced MSC osteogenic differentiation more effectively than higher surface potentials (−76 mV),^49^ supporting the idea that higher stimuli do not necessarily lead to greater differentiation, and that small differences can be critical for the cells. Considering that MC3T3-E1 preosteoblasts present a resting membrane potential of approximately −30 mV to −40 mV,^50^ even small variations in the applied electrical stimulus may modulate voltage-gated ion channels, including calcium channels, thereby influencing the balance between proliferation and differentiation. It is also important to consider that, in addition to the electrical output, the two bioreactor systems applied fundamentally different mechanical cues to the samples. In S-B, the films undergo cyclic deformations in the micrometer range, directly straining the surface and adherent cells. In contrast, the V-B produces mainly oscillatory motion with minimal actual sample deformation and, therefore, with slight surface strain variations.

In parallel, flow cytometry analysis of the cell cycle was carried out on the cells exposed to 48 h of stimulation to further investigate the effect of the different stimuli on cell proliferation behavior. As shown in Figure 5b, MC3T3-E1 cells cultured in GM for 3 days under static conditions exhibited 68.83% of cells in the G0/G1-phase, 10.11% in the S-phase, and 20.81% in G2/M. In contrast, cells cultured under dynamic conditions showed different distributions depending on the type of mechanical stimulus. In the vibration bioreactor, 69.48% of cells were in the G0/G1 phase, 7.08% in the S-phase, and 22.97% in G2/M, suggesting a slight promotive effect on DNA synthesis and cell division. Comparatively, a similar effect was expected for the stretching bioreactor with 113 μVpp mm^–2^ and 227 μVpp mm^–2^ of stimulation. However, the collected cells showed 79.25% and 80.07% in the G0/G1-phase, 3.14% and 2.9% in the S-phase, and 16.74% and 16.27% in G2/M (Figure 5b), respectively. This indicates that a higher electrical stimulus (113 and 227 μVpp mm^–2^) induces cell cycle arrest in G0/G1, reducing DNA synthesis and division, which may suggest a shift toward a more differentiated state rather than active proliferation. Interestingly, despite the high percentage of cells in G0/G1 under the 113 μVpp mm^–2^ condition, lower metabolic activity was observed. This may indicate that, rather than progressing toward differentiation, the cells could be entering a state of reduced metabolic activity or stress-induced arrest. This apparent discrepancy highlights the need to further investigate the threshold at which mechanoelectrical cues transition from supporting proliferation to initiating differentiation. The contrasting mechanical loading regimes of the two bioreactor systems may also help explain these patterns: vibration provides a gentler mechanical environment that supports cell cycle progression and maintains a higher proportion of cells in the S and G2/M phases compared with stretching. In fact, stretching imposes cyclic deformations, increasing cytoskeletal tension and activating mechanotransduction pathways such as MAPK and YAP/TAZ, which are associated with osteogenic differentiation. This shift is reflected in the higher proportion of cells arrested in G0/G1 and the reduced proportion in S phase, indicating decreased proliferative activity in favor of lineage commitment. Chan et al. reported that low-intensity vibration modulates cell proliferation through mechanosensitive pathways, including integrin/FAK/AKT, β-catenin, and YAP, thereby influencing cell cycle progression in a magnitude- and frequency-dependent manner,^34^ supporting the notion that vibration-based stimulation in our study favored proliferation under specific electrical output conditions. These results support the values obtained in the cell viability (Figure 5a). A qualitative analysis of cells was also carried out by cell staining with FITC and DAPI. MC3T3-E1 cells revealed a normal morphology in all cases (Figure 5c).

#### 3.2.2 MC3T3-E1 Culture in Differentiation Medium

The ability of the materials to promote osteogenic differentiation and the effect of the different stimuli on this process were analyzed by culturing preosteoblast cells on the same polymer, PVDF P+, under static and dynamic conditions. After 4 days of culture, the intracellular calcium level was determined (Figure 6) using Fluo-4 AM, a dye that only becomes fluorescent after entering the cell.

**Figure 6:**
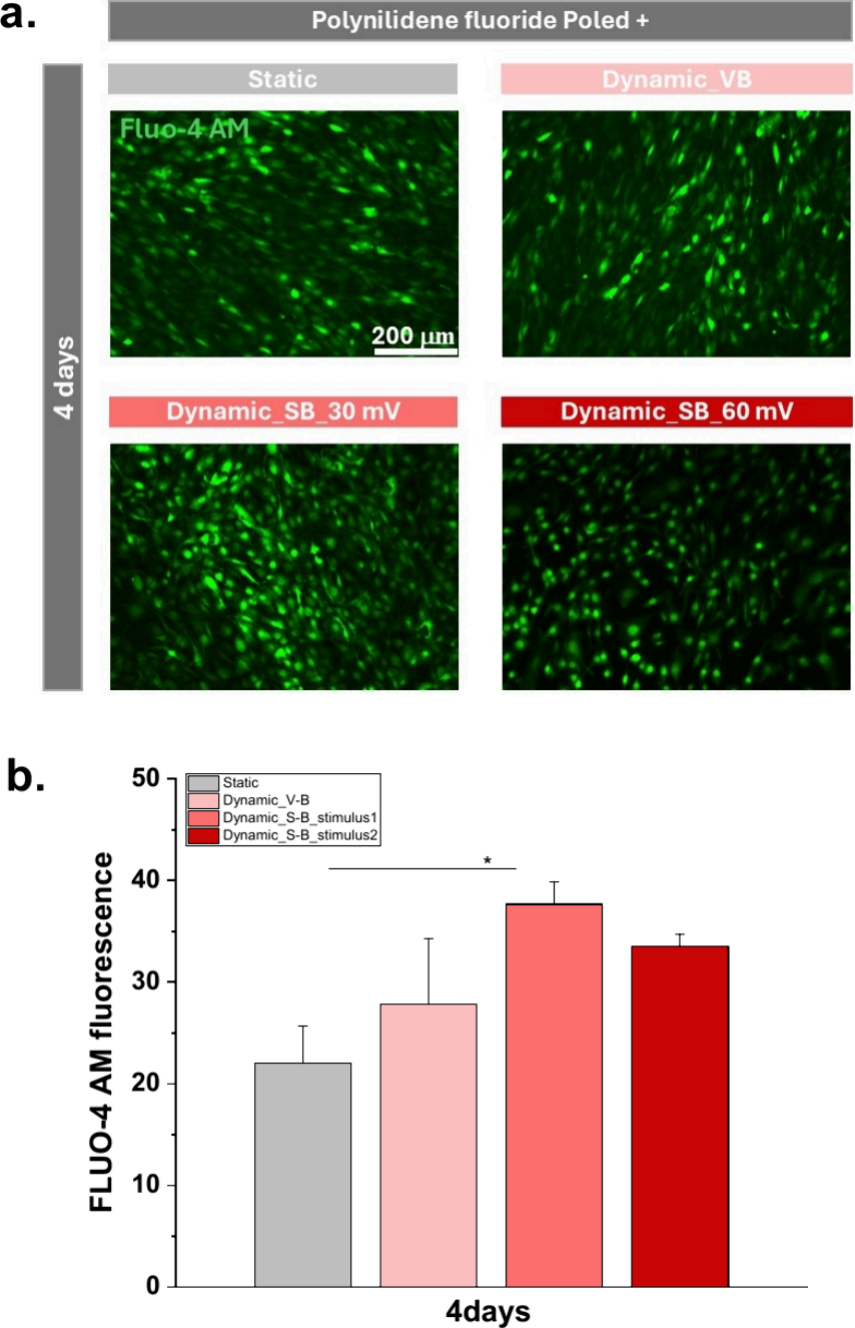
(a) Intracellular Ca^2+^ signaling in MC3T3-E1 cells was examined by loading Fluo-4 AM Ca^2+^ indicator on day 4 after initiating differentiation. Scale bar = 200 μm. (b) Quantification of the fluorescence of [Ca^2+^] in all the parameters studied with PVDF P+ film. Graph bars are represented as mean ± SEM. Significance values: (*) *p* < 0.0332.

Figure 6a shows that mechanoelectrical stimulation induces the highest intracellular calcium concentration in cells on PVDF P+ samples when compared to static conditions, 4 days after starting osteogenic differentiation. This is further confirmed in Figure 6b, where the fluorescence intensity measured with FLUO-4 is clearly higher under dynamic than static conditions, supporting the results observed microscopically.

Figure 6b shows that higher mechanoelectric stimuli (113–227 μVpp mm^–2^) lead to no significant differences between the two conditions applied using the stretching bioreactor, showing an increase in calcium signaling, likely through the activation of mechanosensitive ion channels such as Piezo1 and TRPV4.^51,52^ Intracellular calcium, whether from extracellular influx or released from intracellular stores such as the endoplasmic reticulum, activates key signaling pathways that control osteoblast maturation.^53^ This second messenger is particularly important in early differentiation stages, where it modulates the activation of RUNX2, a transcription factor essential for osteoblast lineage commitment. Additionally, calcium influences the expression of alkaline phosphatase (ALP), an enzyme critical for hydrolyzing phosphate-containing compounds, providing the necessary inorganic phosphate for hydroxyapatite deposition.^54^ Previous studies demonstrated that mechanical stretching stimulation promotes intracellular calcium signaling, reinforcing the importance of this pathway in osteogenic differentiation pathways,^28^ and supporting the results obtained in this study. This interpretation is further supported by evidence showing that different types of mechanical stimulation such as compression versus stretching, elicit distinct calcium signaling responses in osteoblasts, involving different sources and magnitudes of calcium mobilization.^55^ This fact confirms the differences between V-B and S-B used in this study. Moreover, the observed differences between the two stretching intensities in our study may be explained by recent findings indicating that higher mechanical strain can regulate NCX1 expression, leading to calcium efflux and a consequent reduction in intracellular calcium, potentially influencing the balance between proliferation and differentiation.^56,57^ Cyclic stretching, unlike vibration-based stimulation, induces a rapid and transient increase in the intracellular calcium concentration ([Ca^2+^]) through both extracellular influx via stretch-activated channels and release from intracellular stores. As demonstrated by Danciu et al.,^58^ this calcium surge activates downstream signaling pathways, including PI3K/Akt and JNK/SAPK, which are associated with promoting osteogenic differentiation. This mechanism contrasts with vibration, where calcium dynamics are typically more modest, favoring proliferation, rather than initiating the differentiation process.

In order to assess osteoblast differentiation, the alkaline phosphatase activity and alizarin red staining (ARS) were assessed (Figure 7).

**Figure 7:**
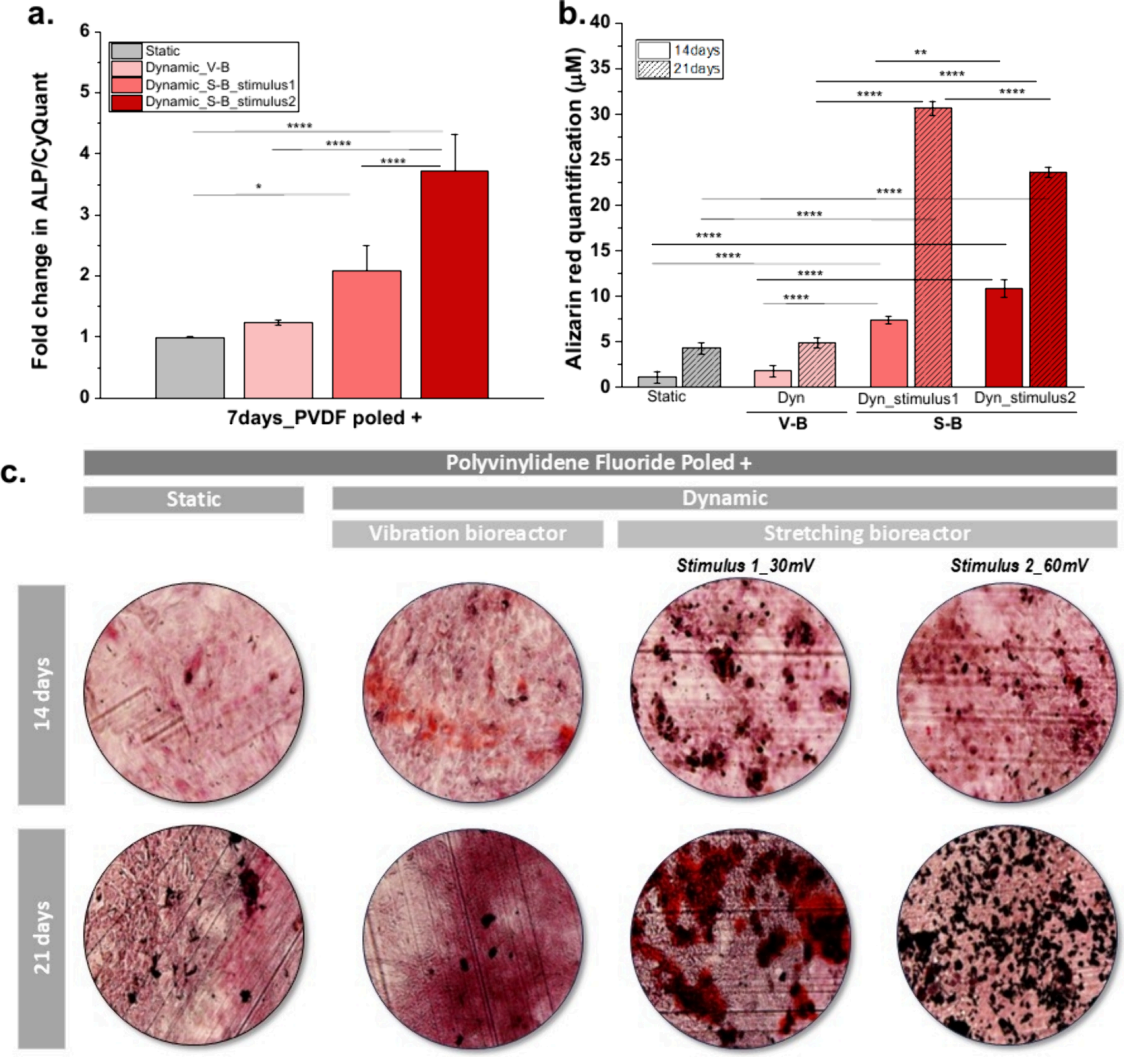
(a) ALP activity at 7 days post-initiating differentiation, (b) quantification of mineralization from alizarin red staining, and (c) qualitative analysis of ARS at 14 and 21 days post-initiating MC3T3-E1 preosteoblast differentiation for PVDF P+ under static and dynamic conditions (3 different conditions). All data are normalized to untreated cells cultured on PVDF P+ under static conditions. Graph bars are represented as mean ± SEM. Significance values: (*) *p* < 0.0332, (**) *p* < 0.0021, (***) *p* < 0.0002, and (****)*p* < 0.0001.

Mechanical stimulation of the cells by stretching significantly increased ALP activity at 7 days post-initiating differentiation (Figure 7a) over 2.1 ± 0.4-fold and 3.7 ± 0.6-fold with an electrical stimulus of 113 and 227 μVpp mm^–2^, respectively, when compared to static conditions. This reveals that ALP expression can be further enhanced when the combination between stretching mechanical stimulus and higher electric stimulus with approximately 227 μVpp mm^–2^ was applied. Contrarily, with a vibration bioreactor (vibration stimulus) and lower electric stimulus (63 μVpp mm^–2^), the ALP expression is lower than the other ones at the same time point of differentiation (1.2 ± 0.04-fold). This enhanced ALP activity under higher mechanoelectrical stimulation conditions is consistent with the earlier observed increase in intracellular calcium levels. Calcium ions, acting as second messengers, contribute to the activation of key osteogenic markers such as RUNX2 and ALP.^59^ Therefore, the calcium influx promoted by mechanical stimulation through the stretching bioreactor likely plays a central role in initiating mineralization processes, which are later reflected in the increased calcium phosphate deposition.^60^ Alizarin red staining was then used to visualize the mineralization potential^61^ of PVDF P+ for preosteoblast cells under each condition (Figure 7b and 7c). The quantification of ARS demonstrated a significant increase in mineralization in both static and dynamically stimulated samples, confirming the progressive mineral deposition between days 14 and 21. Under static conditions, a natural increase in calcium deposition was observed (1.2 ± 0.6 μM to 4.3 ± 0.6 μM, respectively), reflecting the standard progression of osteogenic differentiation. However, samples subjected to stretching bioreactor stimulation with varying mechanoelectrical stimuli between 113 and 227 μVpp mm^–2^ exhibited an even greater increase in ARS, indicating an enhanced mineralization process under biomechanical and bioelectrical stimulation by stretching contrarily to the vibration bioreactor that demonstrates just a slight increase. This superior mineralization effect under stretching is likely related to the higher micrometer-scale deformations transmitted to the cells, which not only stimulate mechanosensitive ion channels but also promote cytoskeletal remodeling and upregulation of mineralization-related genes. Buckley et al.^62^ demonstrated that cyclic mechanical strain (up to 24% elongation at 0.05 Hz) applied osteoblast like cells significantly increased ALP activity within 48 h, indicating an early shift toward a mineralization-competent phenotype. Mechanical strain increased collagen and noncollagenous protein synthesis by day 3 and reorganized the cytoskeleton, with higher levels of vimentin, α-tubulin, and vinculin, indicating stronger focal adhesions. It also shifted protein production toward structural and stress-related proteins, supporting matrix formation. Importantly, mineral deposition was accelerated even without β-glycerophosphate, showing that mechanical strain directly promotes osteogenic progression through combined structural and biochemical effects.^62^

Notably, the most pronounced mineralization at 14 days was observed in the 227 μVpp mm^–2^-stimulated samples (10.9 ± 0.9 μM) with no significant difference with the 113 μVpp mm^–2^ (7.4 ± 0.4 μM) one, contrarily to the 63 μVpp mm^–2^ (1.8 ± 0.6 μM) one, suggesting that higher mechanoelectrical input further enhances calcium deposition. The increase in mineralization across all conditions reinforces the role of external mechanical and electrical cues in accelerating extracellular matrix calcification, likely due to enhanced ionic exchange and osteoblast activity.^63^ At 21 days, the results demonstrated that stretching mechanical stimulation promotes higher differentiation, with significantly higher calcium deposition observed at 113 μVpp mm^–2^ (30.7 ± 0.8 μM) compared to 227 μVpp mm^–2^ (23.7 ± 0.6 μM). An even more marked difference was observed when using the vibration mechanical bioreactor, where a smaller increase in calcium content was detected (4.9 ± 0.6 μM) relative to day 14 values. Figure 7c shows red stained nodules corresponding to mineralization zones, confirming the results obtained in Figure 7b.

To further evaluate the ability of different mechanical and electrical stimuli in promoting osteogenic differentiation, the expression levels of several key osteogenic markers were investigated using qPCR at different time points. The osteogenic differentiation process is regulated by a network of key genes, including ALP (alkaline phosphatase), RUNX2 (runt-related transcription factor 2), OPN (osteopontin), and OCN (osteocalcin), which work in concert to drive bone matrix formation.^64^

The interconnectivity between these genes highlights a well-coordinated regulatory cascade in osteogenesis. RUNX2 initiates differentiation, leading to increased ALP activity, which, in turn, facilitates matrix mineralization. OPN ensures proper control of mineralization, while the OCN finalizes the process by contributing to bone tissue maturation. The synchronized expression of these genes under the influence of different mechanical and electrical stimuli provided by the bioreactors is shown in Figure 8.

**Figure 8:**
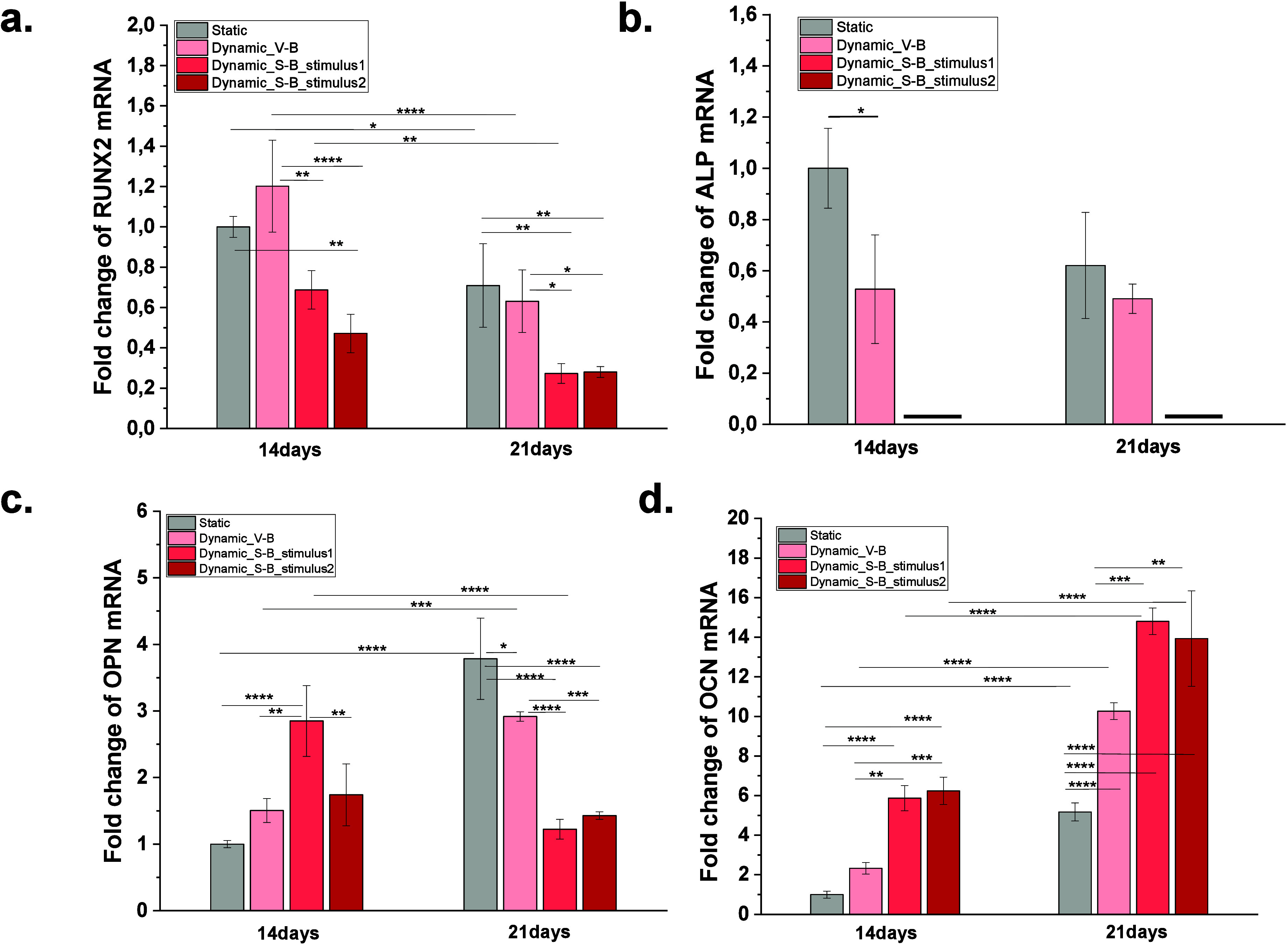
qPCR analysis of osteogenic-related gene expression of (a) RUNX2, (b) ALP, (c) OPN, and (d) OCN in MC3T3-E1 preosteoblasts cultured on neat PVDF P+ , under static and dynamic conditions with different bioreactors. All data are normalized to untreated cells cultured on PVDF P+ under static conditions. Graph bars are represented as mean ± SEM. Significance values: (*) *p* < 0.0332, (**) *p* < 0.0021, (***) *p* < 0.0002, and (****) *p* < 0.0001.

The obtained results indicate that mechanoelectrical stimulation between 113 and 227 μVpp mm^–2^ influences the temporal expression of key osteogenic markers, specifically RUNX2, ALP, OPN, and OCN, suggesting an accelerated differentiation process when compared to static conditions and to lower mechanoelectrical stimulation combined with a vibrational mechanical stimulus during osteoblast differentiation.

RUNX2 is a transcription factor critical for initiating osteogenic differentiation, stimulating preosteoblasts to commit to the osteoblastic lineage. RUNX2 controls the expression of genes involved in the formation of a mineralized matrix such as osteopontin, osteocalcin, and ALP, in the later phases of osteogenic differentiation. In all conditions, its expression typically peaks in the early stages and decreases as cells transition to later differentiation phases. However, under mechanoelectrical stimulation (113–227 μVpp mm^–2^), RUNX2 expression was lower than in static cultures and with a lower stimulus (Figure 8a), indicating that cells had likely progressed beyond the early differentiation phase more rapidly.

Since ALP is a direct downstream target of RUNX2, its expression follows a similar trend. ALP is an early marker involved in the mineralization process by hydrolyzing phosphate-containing molecules to provide free phosphate for hydroxyapatite deposition. Additionally, Kulterer et al. reported that ALP is considered to be an early marker of osteogenesis of osteoblasts cells and is upregulated during the differentiation phase and then downregulated before mineralization.^65^ So, the absence of ALP expression at days 14 and 21 (Figure 8b) suggests that the mineralization phase had already been initiated earlier, reducing the need for ALP activity at later time points.

OPN (osteopontin) plays a crucial role in osteoblast adhesion and regulation of mineralization. It acts as a mineralization inhibitor, preventing premature and excessive matrix mineral deposition. Under static conditions and with lower mechanoelectrical stimulus, the level of the level of the OPN expression remains elevated during the midphase of differentiation, ensuring controlled mineralization. However, under mechanoelectrically stimulated samples with the stretching bioreactor (113–227 μVpp mm^–2^), OPN expression decreased between days 14 and 21 (Figure 8c), suggesting that mineralization was already well underway, reducing the necessity for its regulatory function.

Conversely, OCN, a late-stage osteogenic marker, was upregulated between days 14 and 21 in response to mechanoelectrical stimulation (Figure 8d). OCN is synthesized by mature osteoblasts and is essential for bone matrix stabilization and calcium binding. The increase in the level of OCN expression coincides with the reduction in the level of the level of the OPN, reinforcing the hypothesis that the cells had progressed to the final stages of bone matrix mineralization earlier than in static cultures. This pattern aligns with the mechanical influence of stretching, where higher strain does not only accelerate differentiation but also brings forward the onset of late-stage gene expression, while vibration maintains a slower, more progressive gene expression profile.

In addition to modulating classical osteogenic markers such as RUNX2, ALP, OPN, and OCN, cyclic stretching has also been reported to shift the osteoprotegerin (OPG)/ receptor activator of nuclear factor-κB ligand (RANKL) balance toward an anabolic profile by increasing OPG and reducing RANKL expression in a strain-magnitude-dependent manner, thereby creating a microenvironment that further supports bone formation and matrix mineralization.^66^

Based on all the results obtained, no significant differences were observed between the two electrical stimuli applied through the S-B. However, the mechanical component of the stimulation clearly distinguishes the two bioreactor types, with vibration favoring proliferation-linked gene profiles and stretching promoting earlier shifts toward matrix maturation and mineralization.

Taken together, these findings demonstrate that mechanoelectrical stimulation delivered through the stretching bioreactor accelerates osteogenic differentiation in MC3T3-E1 preosteoblasts, as evidenced by the temporal regulation of key markers such as RUNX2, ALP, OPN, and OCN (Figure 9).

**Figure 9:**
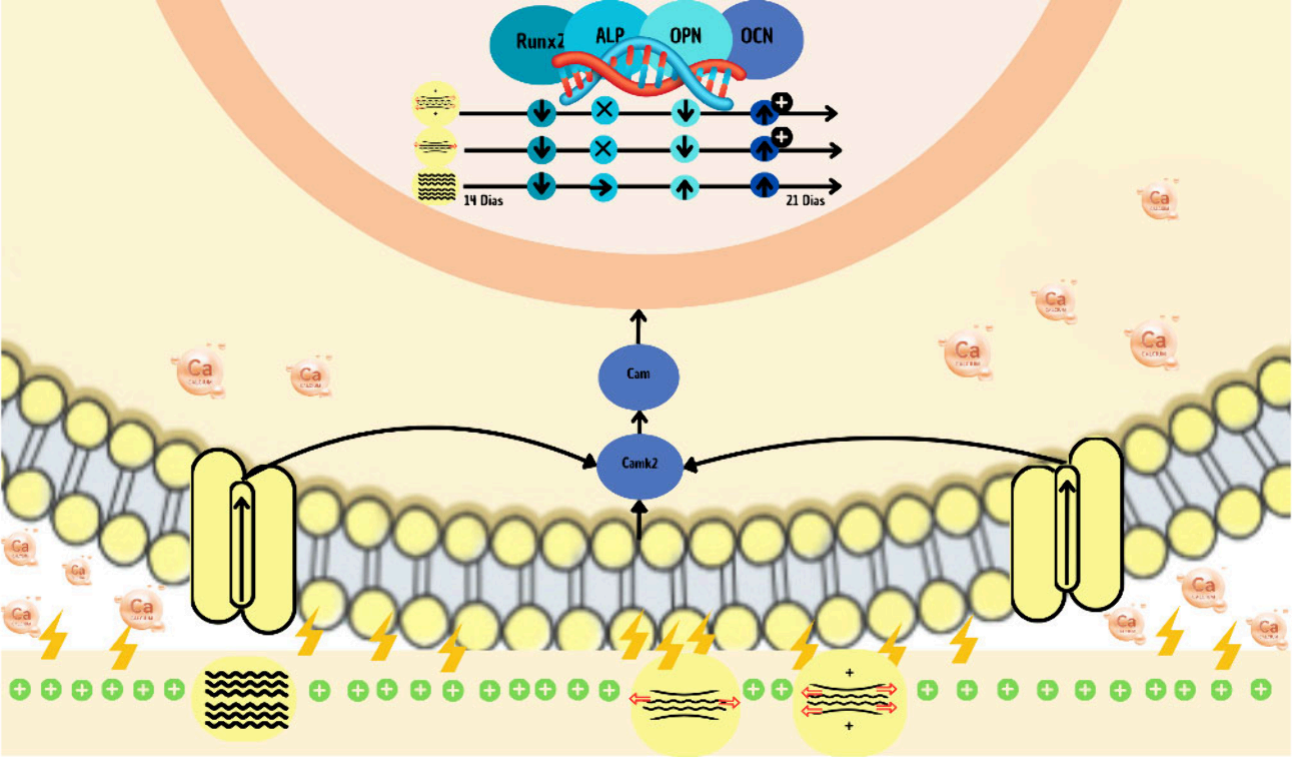
Mechanism of osteogenic differentiation of MC3T3-E1 preosteoblasts cultured on piezoelectric films under vibrational and stretching stimuli.

This accelerated progression suggests that controlled electrical input combined with tailored mechanical cues can effectively modulate the osteogenic process, offering promising perspectives for the development of advanced bioinspired platforms in bone tissue engineering.

## 4 Conclusions

This study demonstrates that piezoelectric biointerfaces can actively regulate osteogenic behavior through the combined action of mechanical and electrical cues. By integrating positively poled PVDF substrates with distinct mechanical stimulation regimes, we show that mechanoelectrical transduction at the material–cell interface plays a decisive role in guiding osteogenic progression.

Cyclic stretching stimulation promoted accelerated osteogenic differentiation, as evidenced by the coordinated temporal regulation of key osteogenic markers and enhanced matrix maturation, while vibrational stimulation favored cell proliferation. These distinct outcomes highlight that cellular responses are governed not only by the presence of electromechanical stimulation but also by the mode of mechanical loading imposed on the cells.

Importantly, the results indicate that different stages of osteoblast development require tailored mechanoelectrical environments, reinforcing the concept that stimulus magnitude and mechanical regime must be carefully optimized to achieve specific biological outcomes. Overall, this work provides new mechanistic insight into the role of piezoelectric biointerfaces as active regulators of cell fate and establishes a versatile platform for the design of smart, stimulus-responsive materials for bone tissue engineering and regenerative applications.

## Supplementary Material



## Data Availability

Data available on request from the authors.

## Notes

Deformation behavior of PVDF specimens under uniaxial tensile loading within the bioreactor operating range (PDF)
