# Supplementary material for: Vibration or Stretch? Distinct Mechanoelectrical Signatures Govern Osteogenic Programming in PVDF
Source: ACS Appl Mater Interfaces. 2026 Feb 9;18(6):9483–96. doi: 10.1021/acsami.5c23327 (PMC12926946; doi:10.1021/acsami.5c23327)
Supplement: Supplementary file 1 [file am5c23327_si_001.pdf]

SUPPORTING INFORMATION

Vibration or Stretch? Distinct Mechano-Electrical Signatures Govern Osteogenic Programming in PVDF

Sylvie Ribeiro[1*], Clarisse Ribeiro[1], Nélson Castro[2], Vitor Correia[3,4], Igor Irastorza[5], Unai Silván[6,7], Senentxu Lanceros-Mendez[1,6,7]

[1.]CF-UM-UP — Physics Centre of Minho and Porto Universities and LaPMET — Laboratory of Physics for Materials and Emergent Technologies, University of Minho, Braga, 4710-057, Portugal.

[2.]Algoritmi Research Centre, University of Minho, Campus de Azurém, Guimarães, 4800-058, Portugal.

[3.]SYSTEC— Research Center for Systems and Technologies- University of Porto (FEUP), Porto, 4200-465, Portugal.

[4.]Faculty of Engineering, University of Porto, FEUP, Porto, 4200-465, Portugal.

[5.]Cell Biology and Histology Department, University of the Basque Country (UPV/EHU), 48940 Leioa, Spain.

[6.]BCMaterials, Basque Centre for Materials, Applications and Nanostructures, UPV/EHU Science Park, Leioa 48940, Spain.

[7.]Basque Foundation for Science, Ikerbasque, Bilbao 48009, Spain.

***Corresponding author:**
Doctor Sylvie Ribeiro, email: sribeiro@fisica.uminho.pt

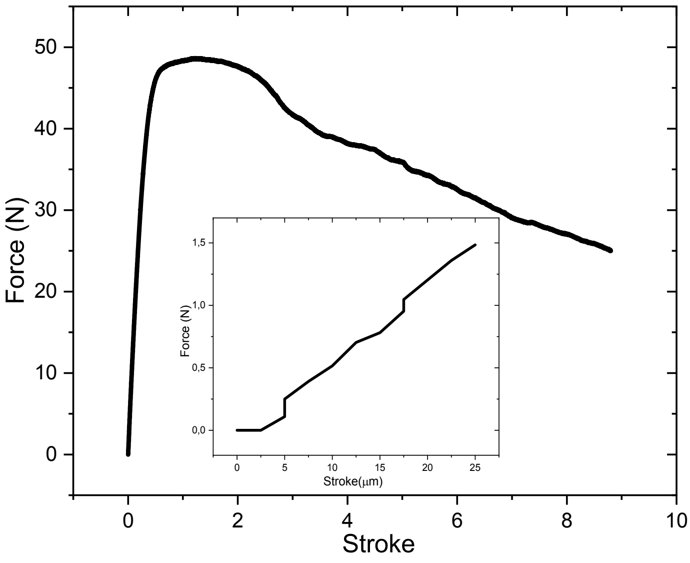

Figure S1. Deformation behaviour of PVDF specimens, under uniaxial tensile load, with application of the bioreactor operating region.
